# Effect of Granule Sizes on Acoustic Properties of Protein-Based Silica Aerogel Composites via Novel Inferential Transmission Loss Method

**DOI:** 10.3390/gels2010011

**Published:** 2016-03-09

**Authors:** Mahesh Sachithanadam, Sunil C. Joshi

**Affiliations:** School of Mechanical and Aerospace Engineering, 50 Nanyang Avenue, Nanyang Technological University, 639798 Singapore; mscjoshi@ntu.edu.sg

**Keywords:** silica aerogel, acoustics, absorption coefficient, composites, inferential transmission loss method

## Abstract

The acoustic properties of the silica aerogel (SA) granules of various sizes from 0.50 to 3.35 mm, distributed into six groups of nominal sizes and measured via a two-microphone impedance tube, are presented. The absorption coefficients of the SA granules were evaluated at ultra- to super-low frequency range from 50–1600 Hz. It was observed that nominal SA granules with sizes of 1.2 mm (AG2) and 1.7 mm (AG3) displayed the best absorption coefficients. When tested with granules filled at 5 cm depth, AG2 and AG3 absorption coefficients peaked at 980 Hz with values of 0.86 and 0.81, respectively. A novel approach to measure transmission loss (TL) by using “inferential” principle is presented. This novel method, named “Inferential Transmission Loss Method” (InTLM), revealed that the average TL, $TL_{avg}$ for both AG2 and AG3 SA granules was 14.83 dB and 15.35 dB, respectively. Gelatin silica aerogels doped with sodium dodecyl sulfate (GSA–SDS) composites comprising of 1.2 mm (GSA–AG2) and 1.7 mm (GSA–AG3) granules of various configurations were fabricated and evaluated for absorption coefficients and TL with known traditional acoustic panels. The results showed that GSA–AG3 had a better absorption coefficient over other configurations for the same corresponding thickness reaching the peak of 0.6 from 1300 to 1450 Hz with $TL_{avg}$ between 10.7 and 20.3 decibels. The four-layered GSA–AG2 and GSA–AG3 composites showed exceptionally high absorption from 500 to 800 Hz suitable for narrow band applications. Lastly, the “InTLM” was matched with the sound meter measurements, with high accuracy between 0.3 and 3.2 dB for low-frequency testing (50–1600 Hz).

## 1. Introduction

The earliest aerogels were made from silica [1,2,3]. In this modern era, technological advancement has made it possible to make aerogels from various materials as reviewed by Koebel *et al.* [4]. Alumina aerogels—which have a rust-like color—synthesized via hydrated aluminum salts and aluminum alkoxides exhibited tremendous potential for high-temperature storage systems and catalysts [5]. Carbon aerogels are used as super-capacitors in electronic circuits and battery-powered portable devices providing bridging power for days [6]. SAs have been studied as a super-insulator used in embedded garments for cold water diving by the US Navy [7]. Similar work by Erik *et al.* [8] to determine the performance of an aerogel blanket coupled with synthetic foam revealed having 35% greater thermal resistance compared with underwater pipeline insulation. Aerogels made from plants cellulose fiber extract have shown to be flexible, transparent and possess good mechanical toughness [9]. Hybrid aerogels made as a result of combining two or more constituent materials are quickly creating a niche in science for specific applications. Carbon nanotubes–graphene aerogel composites decorated with sodium alginate have potential application in heavy metal ion detection [10]. Aerogels made from chalcogens—called chalcogels—originate from the column of elements on the periodic table beginning with oxygen, such as sulfur, selenium, cadmium and platinum. Scientists have shown that chalcogel preferentially absorbs heavy metals and pollutants such as mercury and lead from water [11]. AeroSand, a ceramic-based core material using resorcinol-formaldehyde (RF) aerogel as binder for foundry sand [12] is a current example of an industrial application of the aerogels. Recent works by Hong *et al.* [13] on synthesizing silica aerogel with freeze cast porous zirconia ceramics revealed high compressive strength with reasonably low thermal conductivity ranging from 0.041 to 0.098 W/(m·K). The varied applications in several industries from building construction to di-electrics in integrated circuits are explicitly detailed in a review paper by Gurav *et al.* [14].

Although the prospects of aerogels are very positive, high production costs seem to limit these varieties of aerogels for commercial and business use. The most practical use of aerogels now is thermal insulation. Insulation materials made from silica aerogels are gaining popularity in the United States and the coldest regions of Europe. Besides thermal insulation, aerogels are also used for acoustic and optical insulation in the buildings [15,16]. The global aerogel market was valued at $221.8 million following extensive coverage by Allied Market Research [17] in 2013. It is estimated that it will reach approximately US$1896.6 million by 2020 with a reported Compound Annual Growth Rate (CAGR) of 36.4%. The report evaluated that recyclability, reusability and fire protection will be the notable key drivers in the commercial success of aerogels. Another extensive review on aerogel-based thermal superinsulation by Koebel *et al.* [4] highlighted the above on the various type of aerogel-based products for industrial applications from marine to aerospace. They predicted that the aerogel-based thermal super-insulator market will grow more rapidly than the “conventional” insulators for at least a decade up to the point when markets will begin to saturate. The main drawbacks for aerogels are the high cost of production and the ever-changing global economic conditions. Even so, with advancing methods and technology, the production cost of aerogels is expected to reduce from US$4000 to $1500 per cubic meter [4]. 

Monolithic silica aerogels (SA) synthesized via sol–gel techniques with associated super-critical or ambient pressure drying [18,19,20] are well known to possess porosity of up to 99% [1] with extremely low thermal conductivity and ultra-low density [2,15]. The unique and complex morphological nano-structure of SA contributes hugely to the drastic reduction of conductive and convective gas transport through their pores. This was illustrated in detail from our previous work on the thermal conductivity variations of the CNT-doped SA composites [21]. The nano-pores’ ability to suppress the transport phenomena has similar effects on acoustic attenuation. In recent years, there has been growing interest in the acoustic properties of monolithic SA [22,23]. Sound waves through the nano-pores of SA propagate in the order of 100 ms^−1^ [23], which is about one-third of the velocity of sound in air. Gross *et al.* [24] perceived that the lowest sound velocity achieved is in the range of 80–100 ms^−1^ for non-evacuated SA with a density of approximately 20 kg·m^−3^. However, monolithic SAs are more expensive to produce than the commercially available granular ones. Granular SAs are the cheaper alternative produced on a large scale industrially. Acoustic performance of SA granules previously reported by Forrest *et al.* [25] achieved sound velocity of approximately 60–70 ms^−1^. They reported that the acoustic transmission loss (TL) of SA granules of two sizes, 80 μm and 3.5 mm, via the impedance-matching technique was evaluated to be at least 10 dB higher than glass wool of the same thickness [25]. On field investigations by Cotana *et al.* [26] on a glazing system filled with aerogel granules for thermal, acoustic and lighting performance of a prototype building revealed high correlation amongst the three properties. The promising results further elevate the diverse economic applicability of SA for various sectors and industries as emphasized by Buratti and Moretti in the book titled *Nanotechnology in Eco-Efficient Construction* [27]. The acoustic properties of SAs are greatly influenced by various parameters such as: (a) different chemical reactions during gelation stage of aerogel formation [28]; (b) gas pressure and Young’s modulus of the SA skeleton structure [24]; and (c) the change in the ratio characteristics’ impedance between the medium and the absorption material that consequently determines the variation in the magnitude of reflection and transmission wave [29]. Besides these factors, the type of reinforcement can also significantly enhance the acoustic absorption of SA granules as revealed by Riffat *et al.* [30].

Several methods have been used in determining the TL of an acoustic material. Vigran *et al.* [31] used two different approaches using the full transfer matrix (TM method) and another based on the wave-field decomposition method. However, to implement both techniques, it is necessary to use the four-microphone impedance tube. Smith and Parrott [32] proposed the use of surface methods in determining the acoustic properties—in this case, the propagation constant, by exploiting the changes in surface impedance of specimens. However, this method requires unwrapping of phase information which resides in the imaginary part of the propagation constant to be carried out before any subsequent calculations can be done. In addition, the two-thickness method mentioned previously is more inclined towards well-behaved impedance data where the extraction can be easily automated [33]. 

In our previous works, we developed a low-cost binder-treated silica aerogel composite using gelatin as the main binder; it exhibited high strain recovery with mechanical properties superior to those of SA [34,35], accompanied by super-insulation performance [21]. The present work is an extension from our previous works to study the effects of the SA granule sizes on the acoustic properties of the GSA–SDS composites with the following objectives in mind. First, the acoustic properties of the SA granules of various sizes from 0.50 to 3.35 mm, distributed into six groups of nominal sizes and measured via two-microphone impedance tube, are presented. In addition, GSA–SDS specimens comprising of 1.2 and 1.7 mm SA granules are fabricated and compared with SA granules for variations in acoustic behavior. Second, a simplified novel approach to measure TL by “inferential” principle is proposed and the results are validated with the sound meter measurements. Third, a comparative study of the GSA–SDS acoustic absorption coefficient with traditional absorption material is carried out.

## 2. Results

### 2.1. Silica Aerogel (SA) Granule Optimization

**Figure 1:**
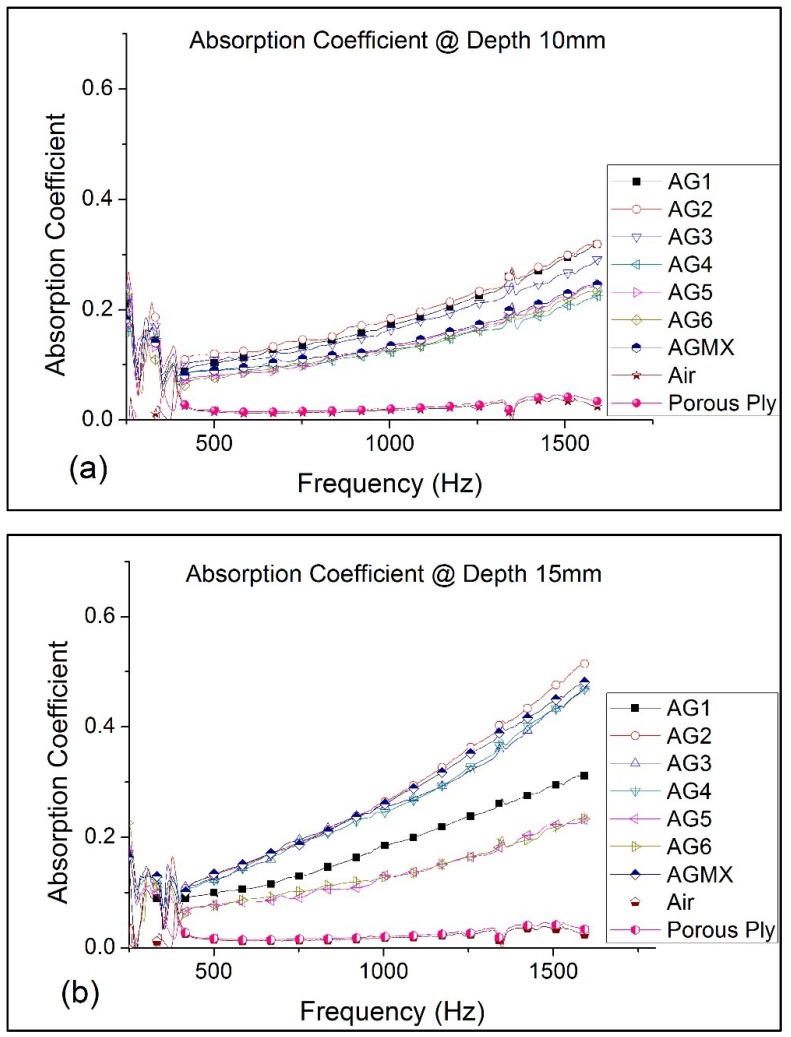
Sound absorption coefficient of silica aerogel (SA) granules (**a**) at 10 mm depth and (**b**) at 15 mm depth.

The two-microphone impedance tube determines the ratio of absorption coefficient and complex reflection coefficient based on the transfer function between the two microphones. The integrated PULSE software that calculates the various data ensures that the sum of the two coefficients shall always conform to unity regardless of the position of the rigid wall. Figure 1 shows the absorption coefficients of the various SA granules for two thickness. The plots in Figure 1b show that AG2 and AG3 have the best absorption coefficient of 0.52 and 0.48 respectively as compared to the other sizes when measured with 15 mm thickness. The absorption coefficients were lower at 0.32 and 0.29 respectively when the thickness was reduced to 10 mm as shown in Figure 1a. It is no surprise that the mixed granules, AGMX, for which 70% of the composition come from the two sizes mentioned, has an absorption coefficient which is close to both AG2 and AG3. The porous ply shows negligible absorption similar to air. Table 1 lists the absorption coefficients abstracted from Figure 1 at the center and maximum frequency.

The absorption coefficient values at ultra-low frequencies from 50 to 200 Hz show erratic response for all the SA granule sizes. One possible reason is that the super-positions of incident and reflected waves at very low frequencies create a region of maximum particle velocity. This usually occurs at one-quarter wavelength, a design reference amongst acoustics engineers who manufacture noise attenuation panels. For a porous material like SA, the absorption is achieved by impeding air particle movement or vibrations and it is most effective in the region of the wave with maximum particle velocity. Thus, the minimum thickness is inversely proportional to frequency and can be written as $\Lambda \;=\frac{c_{max}}{4freq}\;$. This equation suggests that, for a frequency of 200 Hz, the minimum thickness of 40 cm is required to absorb the noise. On the contrary, the minimum thickness is approximately 5.2 cm for higher frequency at 1600 Hz. In Figure 1a,b, similar behavior is observed from 200 to 250 Hz where all the SA granules had one distinctive absorption peak before declining. This is analogous to Helmholtz resonators where the pressure perturbations at the neck produces large velocities into pores of the SA granules. These large velocities in a narrow passage result in considerable viscous dissipation producing high attenuation experiencing resonance [36,37].

**Figure 2:**
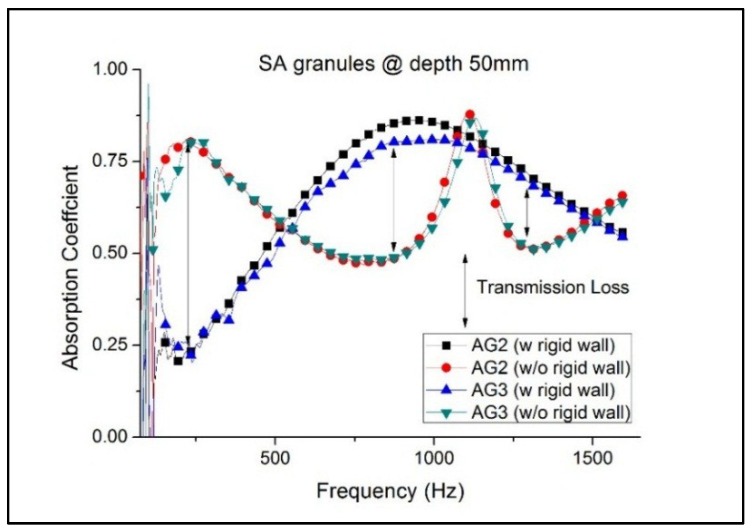
Comparison of AG2 and AG3 granules’ absorption coefficients at 50 mm thickness (with and without rigid wall).

The SA granules have pore sizes in the range of 20 nm. However, the absorption coefficients are varied across the various granule sizes. The absorption coefficient tends to decrease with the increase in granule size. The larger granules AG4—AG6 registered lower absorption coefficients than the AG1—AG3. There is a greater amount of space and voids between the larger granules for a given volume and this could lead to lower acoustic attenuation behavior as shown in Figure 1. On the other hand, the smaller granules tend to be more compact, and having higher silica content for the same volume results in higher packing density. It also helps that the higher silica content contributes to the tortuous path for the sound waves to propagate through the nano-pores of the SA granules. The best absorbing SA granules of 1.2 and 1.7 mm (AG2 and AG3) with specimen thickness of 5 cm are further evaluated for the quarter-wavelength theory for optimization. The absorption coefficients and regions of transmission loss of AG2 and AG3 granules of 5 cm thickness are plotted in Figure 2. The absorption coefficient between the two granule sizes is marginal with 1.2 mm granule size having a better response. However, they both showed similar response peaks (with rigid wall) at 980 Hz with values of 0.86 and 0.81 for AG2 and AG3 granules, respectively. Likewise, the responses from both granules are also similar without the rigid wall.

### 2.2. Transmission Loss of SA Granules

The absorption coefficient, when tested without the rigid wall, peaked at low frequencies from 100 to 300 Hz for both the AG2 and AG3 granules, thereby suggesting part of the pressure wave having been transmitted as loss. Between 300 and 950 Hz, the absorption coefficient begins to decrease, thus indicating that more waves are being reflected. However, on the contrary, the absorption coefficient with rigid wall is seen increasing for the same range. Again, the difference in the responses shows that transmission loss is evident. Similarly, the difference in complex reflection coefficient, although small, also contributes to the ratio of transmission loss. The absorption coefficient of the SA granules generally stabilized at lower frequencies with increasing thickness. However, increase of thickness does not necessarily increase the absorption at higher frequencies (>1000 Hz), where decline is observed for both AG2 and AG3 granules as compared to Figure 1. 

**Figure 3:**
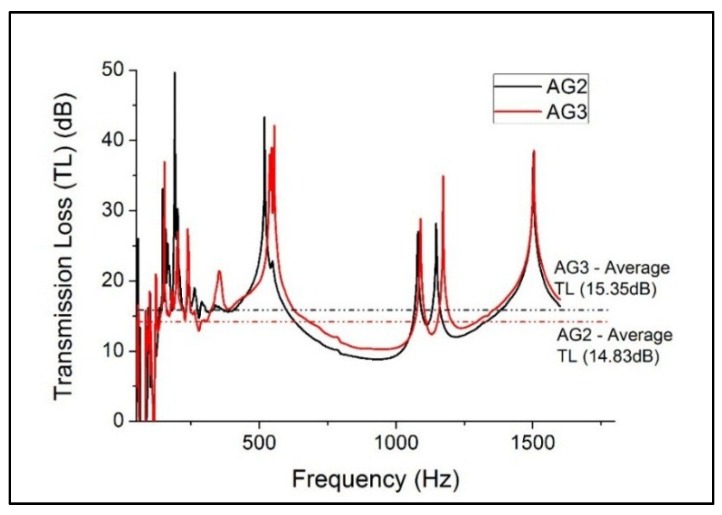
Evaluation of TL for AG2 and AG3 via proposed “Inferential Method” using two-microphone impedance tube.

The proposed “InTLM” (Inferential Transmission Loss Method) from Equations (7) and (8) are used in determining the transmission loss (TL) of the two SA granules as shown in Figure 3. The average TL for AG2 and AG3 granules is 14.83 dB and 15.35 dB respectively. The “Inferential Method” results showed similar findings reported by Forest *et al.* [25] who presented that the TL is lowered by 15 dB for a frequency range from 300 to 1700 Hz for SA granules. The SA granules in Figure 3 showed that TL has several down-peaks for frequencies up to 500 Hz. The TL starts to reduce from 500 to 1100 Hz which corresponds to increase in absorption and reflection coefficients as shown in Figure 2. Subsequently, the SA granules experienced another two down-peaks for frequencies above 1100 Hz. Based on the results obtained in Figure 2 and Figure 3, the AG2 and AG3 granules are selected to fabricate the GSA composite to determine the variations in the absorption coefficient and transmission loss.

### 2.3. Acoustic Performance of Gelatin Silica Aerogels Doped with Sodium Dodecyl Sulfate (GSA–SDS) 

The acoustic absorption coefficients and the TL of the GSA–SDS composites under various configurations are shown in Table 2. 

**Figure 4:**
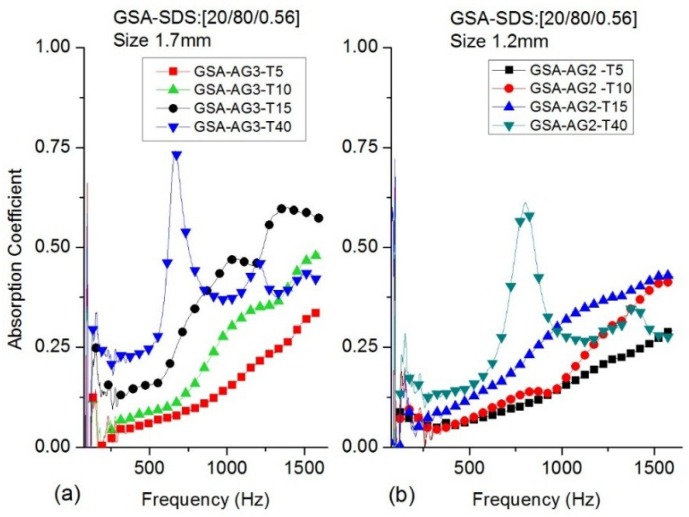
Absorption coefficients for GSA–SDS composites thickness T5–T40 (**a**) size 1.7 mm; (**b**) size 1.2 mm for frequency (50–1600Hz).

Figure 4 shows the absorption coefficient values of GSA–SDS composites for the various SA granule size and specimens’ thickness range listed in Table 2. The absorption coefficients for GSA–AG3 show better response than GSA–AG2. Interestingly, the response reported earlier in terms of SA granules was the reverse. The GSA–AG3–T15 composites show better absorption at lower frequencies than the GSA–AG2–T15 blocks. It steadily rises to a maximum of 0.6 from 1300 to 1450 Hz before declining to 0.57 at 1600 Hz. Generally, the GSA–SDS composites also showed better absorption for the same thickness at the center frequency and maximum frequency as compared to SA granules as shown in Table 1. The variations in densities between the SA granules and GSA–SDS composites, in general, are negligible between 0.003 and 0.005 g/cm^3^. This indicates that the gelatin in the composite contributed to a very marginal increase to the overall density. The gelatin foam that encompassed the SA granules would have certainly provided additional obstacles to the sound’s path by introducing more voids between the granules and thus an increase in the absorption coefficient. 

The four-layered GSA–SDS composites (GSA–AG2-T40 and GSA–AG3-T40) of 40 mm thickness are evaluated for absorption coefficient. The response of both the granule sizes is similar. However, the frequency at which the peak occurred is different. The composites with AG3 granules registered the maximum absorption of 0.74 at 670 Hz. In contrast, for the composites with AG2 granules, the maximum peak 0.61 resided at the center frequency of 800 Hz. However, both the four-layered composites showed rapid rise from 500 to 800 Hz in absorption as shown in Figure 4a,b, which is different from the SA granules that have a gradual slope from 500 to 1100 Hz. The results showed interesting behavior in the response between the single block layer and the four-layered composite. The fourlayered GSA–SDS composites have better absorption at lower frequencies as compared to the single GSA–SDS blocks and *vice versa* at higher frequencies. 

**Figure 5:**
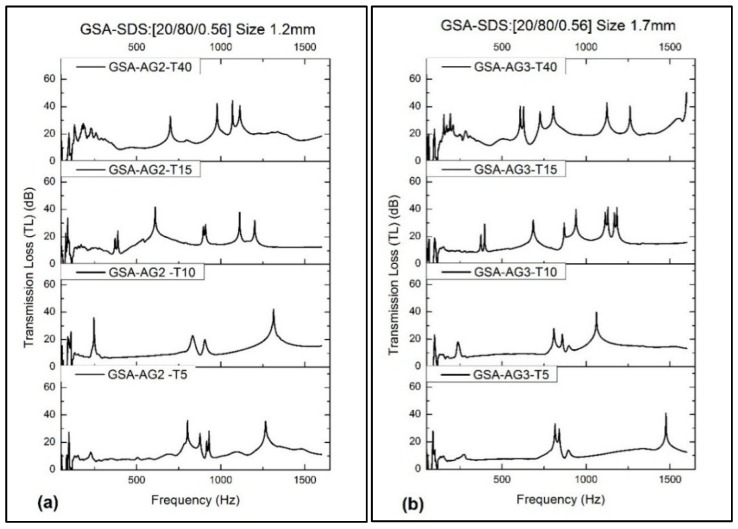
Transmission loss (TL)for GSA–AG2/AG3 composites’ thickness T5–T40 (**a**) size 1.2 mm; (**b**) size 1.7 mm.

The TL for the GSA–AG2 and –AG3 composites is shown in Figure 5. Generally, the average TL increases with increasing thickness of the composites. As the thickness increases, more peaks start to appear with narrowing bands, especially the four-layered GSA–SDS composites However, the GSA–AG2 and –AG3 composites of 10 mm thickness showed better stability as there are fewer peaks than the rest. In terms of magnitude, there is not much of difference between 1.2 mm and 1.7 mm SA granules and it remains inconclusive as to which is better at reducing TL with the exception of the four-layered GSA–SDS composite. The average TL is between 10.7 and 20.3 decibels (dB).

The “InTLM” in Table 2, when compared with the sound meter measurements, showed high accuracy of 0.3 to 3.2 dB for low frequency testing (50−1600 Hz). A key reason for the high accuracy is due to close matching of data between the sound meter and impedance tube. The data points captured over the entire run in the sound meter last approximately 35 s. Given that the period of capturing one discrete data in sound meter is 20 ms, the sound meter effectively captures about 1750 discrete data over the entire run. Therefore, it is closer to the large tube setup for the frequency range up to 1600 Hz, assuming that one data per Hz is captured in the impedance tube.

### 2.4. Comparative Analysis with Other Traditional Materials

**Figure 6:**
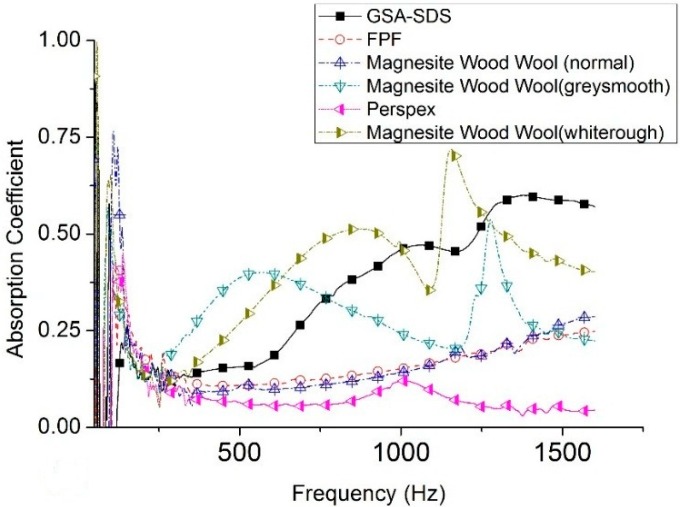
Comparison of absorption coefficients of GSA–SDS with other materials for 15 mm thickness at 50–1600 Hz.

The absorption coefficient performances of the GSA composites and other traditional materials are presented in Figure 6.

The GSA–SDS composite, when compared with acrylic panel, grey FPF and the magnesite wood wool (normal), have superior acoustic absorption throughout the entire range of frequencies. However, when compared with the magnesite-coated wood wools (grey/smooth and white/rough); the GSA–SDS-related composites were inferior at low frequencies from 300 to 750 Hz. After 750 Hz, only the white/rough magnesite wood wool showed better acoustic absorption up to 1150 Hz. Thereafter, the GSA–SDS exhibited superior performance until 1600 Hz. The micro-coating on the wood wool definitely enhanced their absorptive capacity at certain bandwidths as the micro-beads of the ceramic created a tortuous path as compared to the bigger gaps in normal ones. Similarly, if the pore size in SA granules is reduced, debilitating the paths undertaken by the sound waves, the GSA–SDS composites would have better acoustic performance. In addition, if the pores induced in the gelatin network around the SA granules can be reduced or controlled, then chances of better absorption could be realized. 

### 2.5. Acoustic Activity

Acoustic activity [38,39] is defined as the area under the absorption curve normalized over the frequency range and determined as shown in Equation (1) where, f_1_ is the lower frequency at 50 Hz and f_2_ is the upper frequency at 1600 Hz.

(1) $$\alpha_{normalized}=\frac{1}{\left(f_{2}-f_{1}\right)}\;\int_{f_{1}}^{f_{2}}\alpha \left(f\right)\;df$$

**Figure 7:**
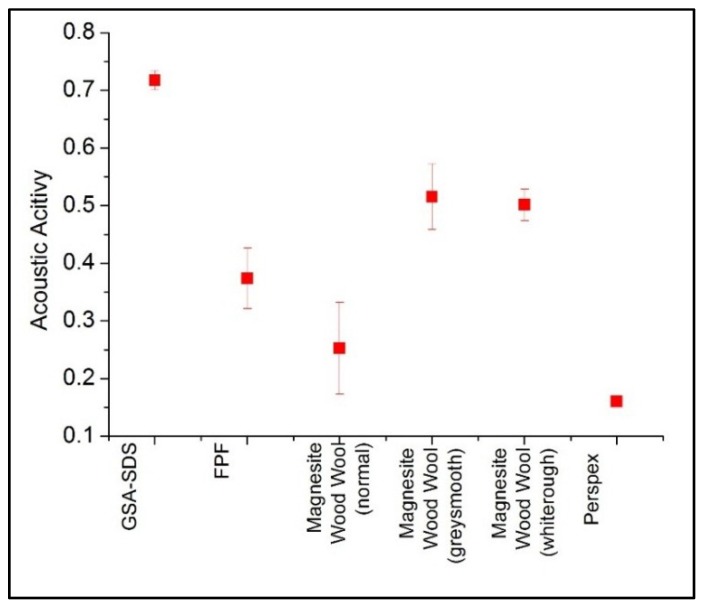
Normalized acoustic absorption coefficient (acoustic activity) of selected materials.

The acoustic activity of the various material experimented in the preceding section has been determined and shown in Figure 7. The values would give a visible and quick identification on the best absorbing materials over the entire range of frequency to be operated. The GSA–SDS composites displayed the highest acoustic activity amongst the materials tested. The two magnesite-coated wood wools are the next highest at approximately 0.5 and undoubtedly Perspex has the lowest acoustic activity. The values showed that GSA–SDS is the best option if one wants to use a material that is capable of maintaining high absorption in a wide frequency range. 

## 3. Conclusions 

There are two key aspects to the study. First, a comparative study on the acoustic absorption coefficient and TL of SA granules of various sizes and GSA–SDS composite are evaluated at low frequency range from 50 to 1600 Hz. It was noted that AG2 and AG3 granules result in the best absorption coefficients, peaking at 980 Hz with the values of 0.86 and 0.81 when tested with 5 cm depth with an average TL of 14.83 dB and 15.35 dB respectively. It was also noted that as a composite block, the GSA–AG3 composites have a better absorption coefficient than GSA–AG2 for the same corresponding thickness, reaching the peak of 0.6 from 1300 to 1450 Hz. Overall, the GSA–SDS composites with 10 mm thickness exhibited superior absorption over the SA granules and better stability in terms of TL. It was revealed that the four-layered GSA–SDS composites’ configuration is suitable for acoustic absorption in narrow band application. The GSA–SDS composites comprising of SA granules are suitable for both as acoustic absorption and acoustic barrier material. In addition, the acoustic activity has shown that GSA–SDS composites have better absorption than other traditional materials over a wider range of frequencies.

The second aspect is the proposed novel “Inferential Transmission Loss method” (InTLM) in determining TL using a two-microphone impedance tube. The approach is a modification to the usual transfer method by inferring the transmission coefficient with and without the rigid wall. The calculated results showed high accuracy from 0.2 to 3.2 dB as compared with sound meter measurements. Thus, the “InTLM” can be applied for 100 mm diameter specimens which uses the large tube in estimating the TL without the need to use a four-microphone impedance tube. Most porous materials absorb incident and airborne sound waves well. A small change in pressure perturbations can generate loud noise which dissipates into heat as it travels through the tortuous path in these materials. On the other hand, absorbers are usually not good as acoustic insulators or barriers, a property that is directly related and proportional to the mass of the wall. The heavier the mass of a wall, the better it acts as a sound insulation, especially against airborne noise. However, GSA–SDS through the experiments have shown to possess a balanced feature both as a sound absorber and a barrier despite being lightweight. In our previous work [34], we had determined the compressive modulus for the GSA–SDS specimens to be in the range of 0.328−0.434 MPa for corresponding densities between 0.067 and 0.070 g·cm^−3^. The sound velocity, c=Eρ′2, propagating through these specimens, would be approximately 69.96–78.24 m/s. The ability of the GSA–SDS composites to retard sound propagation with absorption is significant especially in buildings where lightweight walls are designed for thermal insulation but not for sound insulation. GSA–SDS composites are able to fulfill both criteria to support both as an acoustic and thermal insulation material. Future works shall include *in situ* measurements and field experiments of GSA–SDS-impregnated windows and wall panels.

## 4. Experimental Procedures

### 4.1. Specimen Preparation

**Figure 8:**
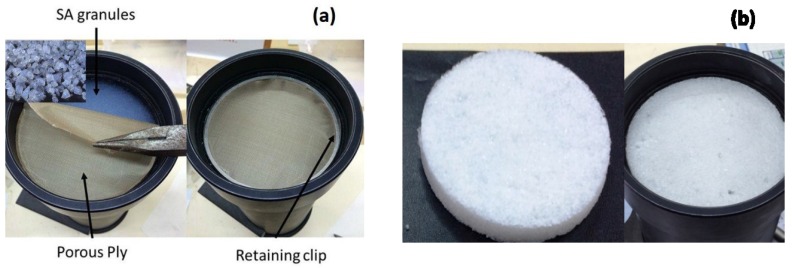
(**a**) Filling of SA granules in impedance tube; (**b**) GSA–SDS specimen.

Figure 8a shows how the SA granules (see insert picture) are prepared for the experiment. First, the acoustic absorption of an appropriate thin porous ply is evaluated. Then, SA granules of various sizes are filled in the impedance tube to the depths of 10 and 15 mm covered with a layer of porous ply held using a retaining clip. The absorption coefficients of different SA granules with the porous ply are determined. Three measurements for each depth size are then taken. The average measurement is then subtracted from the porous ply results to give the absorption coefficient of the SA granules as plotted in Figure 1.

GSA–SDS specimens of 100 mm diameter in varying thicknesses are fabricated via the freeze-drying method described in the same manner as previously reported [21] comprising of 1.2 and 1.7 mm SA granule sizes (GSA–AG2 and GSA–AG3). The composition of GSA–SDS specimen is 20 wt% gelatin; 80 wt% SA; 0.56 wt% SDS as shown in Figure 8b. The preparation for the acoustic measurements for GSA–SDS specimens is the same as SA granules described in the above paragraph.

### 4.2. Transfer Function Method (Two-Microphone)

**Figure 9:**
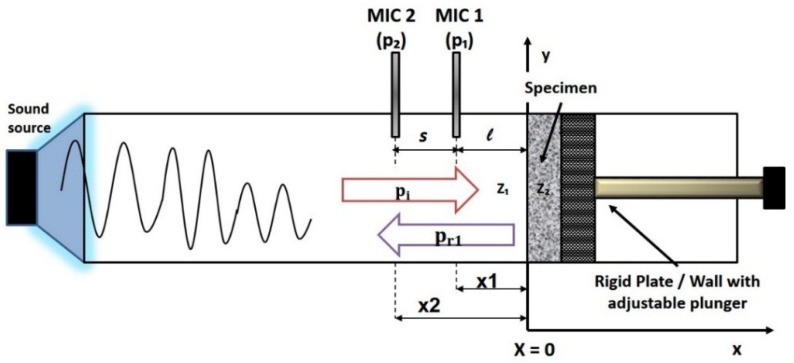
Schematic of plane wave generation for acoustic measurement using two-microphone impedance tube.

Figure 9 shows the generation of plane waves from the sound source within the impedance tube. A source sound, usually a loudspeaker, is mounted at one end of the impedance tube and a specimen of the material under test is placed at the other end. The speaker generates broadband, stationary random sound waves which will propagate as plane waves and reflect after hitting the sample. The decomposition of the stationary sound waves pattern into forward and backward travelling components inside the tube produces a standing wave interference pattern. By simultaneously measuring the sound pressures at two fixed locations (mic 1 and 2) and calculating the complex transfer function, it is possible to determine the complex reflection coefficient, the sound absorption coefficient and the normal acoustic impedance of the specimen. The reference plane (x = 0) is taken as the front surface of the specimen as indicated in Figure 9. The usable frequency range depends on the diameter of the tube and the spacing between the microphone positions. B&K 4206 Impedance Tube Kit (Bruel & Kjaer, Norcross, GA, USA) comes with two types of tubes. The large tube of 100 mm measures for frequency range from 50 to 1600 Hz and the small tube of 29 mm measures the 500–6400 Hz. The scope of work carried out in this study is limited to experiments using the large tube only.

SA granules, as highly porous solids, have the potential to be a sound-absorbent material. Sound absorbers are usually characterized by surface impedance and absorption coefficient. A one-dimensional plane wave in the tube is assumed to be $pe^{j\left(\omega t-kx\right)}$ [40]. The time-dependent term which represents the pressure perturbations in travelling waves with respect to x direction, is eliminated since the microphones are at the fixed location. The total acoustic pressures about the fixed locations at the two microphones, $p_{1}$ and $p_{2}$ can be expressed as follows in Equation (2a) and (2b) as shown below.

(2a) $$p_{1}=p_{i}e^{-jkx1}+p_{r1}e^{jkx1}$$

(2b) $$p_{2}=p_{i}e^{-jkx2}+\;p_{r1}e^{jkx2}$$

where:

- $p_{i}\;$and $p_{r1}$ are the sound amplitude of the incident and reflected pressure respectively.
- k is the wavenumber of the incident sound pressure, therefore $k=\frac{2\pi f}{c}$
- *f* is the frequency and c is the speed of sound.
- x1=−l and x2=−s−l are the distances from the specimen to the microphones 1 and 2, respectively.

The complex reflection coefficient $R_{1}$ is the ratio of the reflected wave to the incident wave. The transfer function H is defined as the ratio between the acoustic measurements of $p_{1}$ to$\;p_{2}$. Equation (2a) and (2b) can be rearranged as shown in Equation (3a) to (3c):

(3a) $$p_{i}=\frac{j(p_{1}e^{jkx2}-\;p_{2}e^{jkx1})\;}{2sink\left(x_{1}-x_{2}\right)}$$

(3b) $$p_{r1}=\frac{j(p_{2}e^{-jkx1}-\;p_{1}e^{-jkx2})}{2sink\;\left(x_{1}-x_{2}\right)}$$

(3c) $$R_{1}=\frac{p_{r1}}{p_{i}}=\frac{(p_{2}e^{-jkx1}-\;p_{1}e^{-jkx2})\;}{(p_{1}e^{jkx2}-\;p_{2}e^{jkx1})\;}$$

Substituting $H=p_{1}/p_{2},$ x1=−l and x2=−s−l will yield the complex reflection coefficient, $R_{1}$.

(4) $$R_{1}=e^{j2k\left(s+l\right)}\times \;\frac{(e^{-jks}-\;H)\;}{\left(H-\;e^{jks}\right)\;}$$

The Equation (4) is the reflection coefficient for the two-microphone transfer function method. When the specimen is backed by a rigid back wall, there will be no transmitted waves and thus by conservation of energy, all the incident waves are reflected and absorbed. The absorption coefficient ($\alpha_{1})$ can therefore be expressed as in Equation (5).

(5) $$\alpha_{1}=1-\;{\left|R_{1}\right|}^{2}=1-R_{r1}^{2}-\;R_{i1}^{2}$$

### 4.3. Inferential Transmission Loss Method (InTLM)

One of the drawbacks of the two-microphone transfer function method is that the absorption coefficient determined may not be a true representation of the material’s characteristics. In the case of a porous material, such as SA, the reflected wave from the rigid wall could contribute to rise in the absorbed energy by the material. To account for this uncertainty, the four-microphone impedance tube setup is usually used to determine the transmission loss (TL) and absorption coefficient [41]. In the absence of additional microphones downstream of the specimen, a sound meter could be used instead to measure the TL of the specimen under test. However, the sound meter picks up discrete transmitted signals at periodic intervals, which could result in a mismatch with the generated signals from the source if not calibrated carefully. 

**Figure 10:**
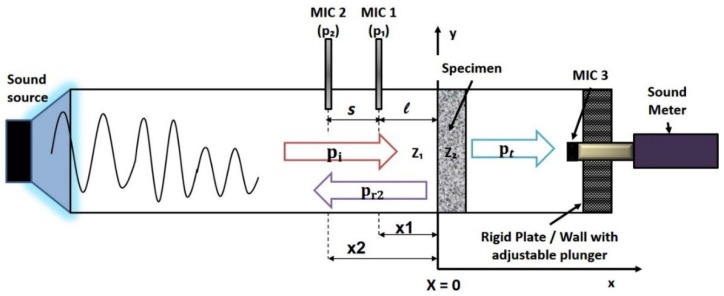
Schematic of “InTLM” experimental setup for transmission loss measurement using two-microphone and sound meter without the rigid wall backing.

In Figure 10, a novel approach to measuring the TL is proposed using InTLM. The incident sound wave is reflected, absorbed and transmitted through the specimen since the rigid wall is moved to the end of the impedance tube instead. Thus, one would expect variations in the absorption coefficients as there is space behind the specimen for the sound waves to propagate through the specimen. The energy conservation would mean that the total acoustic pressures on both sides of the specimen would be the same as shown in Equation (6a). Equation (6b) represents the new absorption coefficient in the absence of the rigid wall backing behind the specimen.

(6a) $$E_{I}=\;E_{R2}+E_{T}+E_{\alpha 2}$$

(6b) $$\alpha_{2}=1-\;{\left|R_{2}\right|}^{2}-\;{\left|T_{r}\right|}^{2}$$

Subtracting Equation (5) from Equation (6b), an inferred expression for transmission coefficient can be derived without the use of four-microphone setup.

$$\alpha_{2}-\;\alpha_{1}=1-\;{\left|R_{2}\right|}^{2}-\;{\left|T_{r}\right|}^{2}-\left(1-\;{\left|R_{1}\right|}^{2}\right)$$

(7) $${\left|T_{r}\right|}^{2}=\left(\;{\left|R_{1}\right|}^{2}-\;{\left|R_{2}\right|}^{2}\right)+\left(\alpha_{1}-\;\alpha_{2}\right)$$

(8) $$TL=10log_{10}{\left|T_{r}\right|}^{2}$$

The expression in Equation (7) shows that the InTLM way of determining transmission loss coefficient,$\;T_{r}$ is simply the sum of the difference between measured reflected coefficients and the absorption coefficients. The change in values of the two terms would then be defined as TL as shown in Equation (8). The word “inferential” often appears in literature across many fields of research. However, it should not be confused with the works of Xiang *et al.* [42,43,44,45,46] that were developed from a Bayesian model-based framework to determine the conditional probabilities correlating with new and supporting evidences. 

### 4.4. Sound Meter Measurements

In order to validate the accuracy of the TL determined via “InTLM,” a sound meter is utilized during the experiments. The adjustable plunger was removed and a sound meter is placed at the end cap. The sound meter is turned on concurrently with the impedance tube. The sound meter captures the noise level detected at a distance of 200 mm from the specimen. The impedance tube generates about approximately 106.3 dB of sound pressure. The difference between the sound meter value and the source is the transmission loss. The sound meter captures discrete data periodically at 20 ms. Thus, the most practical way of validating the results is to compare the average TL between the sound meter and the “InTLM.”

## 5. Material and Equipment 

Commercially purchased hydrophobic SA granules with 99.9% porosity from Cabot Corp (Alpharetta, GA, USA) come in sizes ranging from 0.10–4.00 mm. The aerogel granules were placed in a mechanical sieve shaker consisting of seven sieve sizes and a pan. The granules were sorted according to their particle distribution from the mass distribution in the sieves as reported in our previous work [21]. The average distribution from a total of 10 sample sets is taken. The aspect ratio of the granules is the ratio of the maximum granule size to the minimum granule size for a particular group. In this work, only the sizes ranging from 0.50–3.35 mm are investigated for the acoustic properties as they represent approximately 99% of the overall distribution [21]. The density of the granules is calculated from the mass measured from Mettler Toledo AB-265S/Fact High Precision Balance (Columbus, OH, USA) in volumes of 25–100 cm^3^ at 25 cm^3^ increment. The 100 mm diameter GSA–SDS specimens were weighed on the same balance and the density calculated from the known mass and dimension. Table 3 shows the group classification, sample distribution and their physical properties for each granule size. High-Strength Gelatin from porcine skin (bloom strength 240–270; density ~1.043 g/cm^3^) and SDS (density 0.37 g/cm^3^) were purchased from Sigma Aldrich (Singapore, Singapore). 

**Figure 11:**
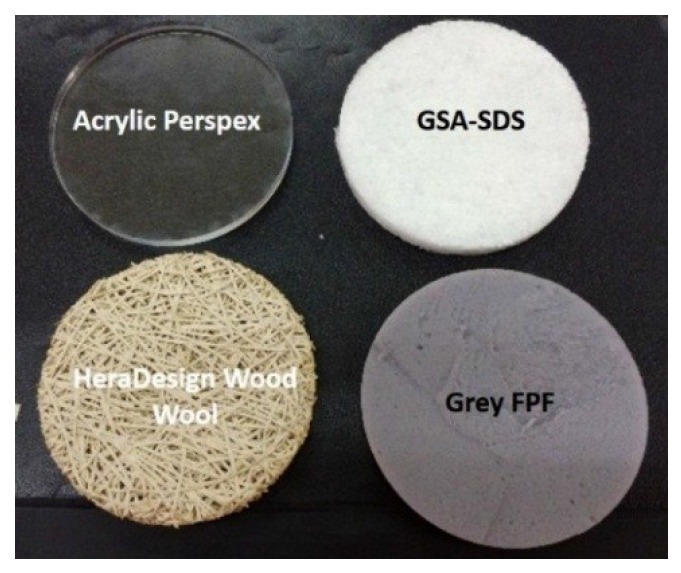
Various types of materials tested.

In addition, the absorption coefficient of GSA–SDS composites were compared with other materials as listed in Table 4. Figure 11 shows the materials evaluated for comparative analysis. The grey flexible polyurethane foam (FPF) and acrylic Perspex were taken from the in-house laboratory. The magnesite wood wools were acquired from Heradesign Inc. (Ferndorf, Austria), a company that manufactures acoustic panels. Three types of panels were tested; (a) normal panel bonded with layers of 2 mm fiber wood wool, (b) same as (a) but coated with a layer of grey and smooth micro-ceramic coating and (c) same as (a) but coated with a layer of white and rough micro-ceramic coating. 

**Figure 12:**
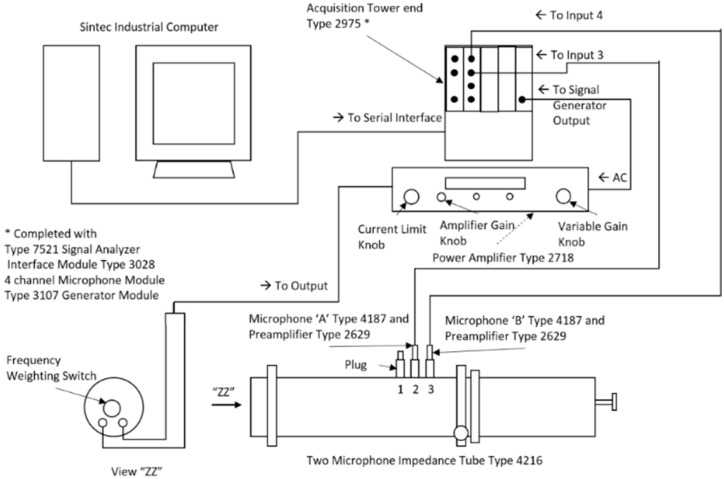
Experimental setup using the B&K Type 4206 Impedance Tube Kit as per ASTM E1050.

Acoustic measurement for the SA granules, GSA–SDS composites and the listed materials above were carried out as per ASTM E1050 [47] using the Bruel and Kjaer (B&K) Type 4206 Impedance Tube Kit (Bruel & Kjaer, Norcross, GA, USA) coupled with Digital Frequency Analysis System (Norcross, Georgia, USA). Random noise or white noise as it is commonly known, is generated by using a B&K generator module Type 3107 that is equipped with four-channel microphone module type 3028 and amplified using power amplifier type 2718 set at 1 ampere RMS with zero-degree phase input/output. B&K Pulse testing program type 7758 is the integrated software that interfaces the signals into experimental data. The experimental setup is shown in Figure 12. 

## Figures and Tables

**Table 1:** Absorption coefficient of SA granules, Air and Porous Ply.

Classification	Absorption Coefficient (10 mm)		Absorption Coefficient (15 mm)	
	800 Hz	1600 Hz	800 Hz	1600 Hz
AG1	0.14	0.31	0.14	0.31
AG2	0.14	0.32	0.20	0.52
AG3	0.14	0.29	0.21	0.48
AG4	0.10	0.23	0.20	0.47
AG5	0.10	0.25	0.10	0.24
AG6	0.11	0.24	0.11	0.24
AGMX	0.11	0.25	0.20	0.48
Air	0.00	0.00	0.00	0.00
Porous Ply	0.01	0.01	0.01	0.01

**Table 2:** Densities, Absorption Coefficient and Transmission Loss of GSA–SDS Composites.

Granule Size (mm)	Density ρ (g/cm^3^)	No. of Layers	Thickness, T (mm)	Frequency (Hz)	Absorption Coefficient	Transmission Loss (dB)	TL(avg) (dB)	Sound Meter TL(avg) (dB)
**GSA–AG2 (1.2 mm)**	0.079	1	5	800/1600	0.11/0.29	26.0/11.2	11.3	11.0
	0.073	1	10	800/1600	0.14/0.41	12.0/15.5	11.7	12.8
	0.071	1	15	800/1600	0.22/0.43	15.2/12.6	14.6	11.3
	0.074	4	40	800/1600	0.61/0.28	15.7/18.6	16.4	15.2
**GSA–AG3 (1.7 mm)**	0.084	1	5	800/1600	0.10/0.34	16.1/12.5	10.7	10.9
	0.074	1	10	800/1600	0.17/0.49	16.9/13.3	11.8	12.6
	0.072	1	15	800/1600	0.36/0.57	11.4/15.8	14.5	13.3
	0.075	4	40	800/1600	0.43/0.42	33.0/42.9	20.3	18.6

**Table 3:** Classification and physical properties of SA granule sizes.

Classification	Size Range d (mm)	Median d (cm)	Density ρ (g/cm^3^)	Distribution %	Aspect Ratio
AG1	0.50 ≤ x < 1.00	0.075	0.0682	2.10	1.98
AG2	1.00 ≤ x < 1.40	0.12	0.0693	7.01	1.39
AG3	1.40 ≤ x < 2.00	0.17	0.0719	63.15	1.42
AG4	2.00 ≤ x < 2.36	0.218	0.0727	19.69	1.18
AG5	2.36 ≤ x < 2.8	0.258	0.0732	5.96	1.18
AG6	2.8 ≤ x < 3.35	0.307	0.0748	1.22	1.19
AGMX	0.10 < x ≤ 4.0	0.200	0.0723	100.0	1.35

**Table 4:** Classification and physical properties of other materials.

Classification	Type	Density ρ (g/cm^3^)
**Grey FPF**	Foam, flexible	0.0068
**Acrylic**	Transparent, rigid, hard	1.2072
**HeraDesign Inc Magnesite wood wool**	Normal	0.4082
	Coated with smooth micro-ceramic layer	0.6395
	Coated with rough micro-ceramic layer	0.5660
